# Acute Pericarditis Occurring Three Days after Intravesical Instillation of Mitomycin C after Transurethral Bladder Tumor Resection in a 64-Year-Old Woman

**DOI:** 10.1155/2018/9130852

**Published:** 2018-02-21

**Authors:** Vineet Meghrajani, Arsalan Hashmi, Shuo Cheng Lin, Zvi Plawes, Shelly Brejt

**Affiliations:** ^1^Department of Internal Medicine, Maimonides Medical Center, 4802 10th Avenue, Brooklyn, NY 11219, USA; ^2^Department of Cardiology, Maimonides Medical Center, 4802 10th Avenue, Brooklyn, NY 11219, USA

## Abstract

We present a 64-year-old woman who developed symptoms of acute pericarditis three days after undergoing intravesical instillation of mitomycin C following transurethral bladder tumor resection. Mitomycin C is a chemotherapeutic agent which acts by alkylation of DNA and is known to be cardiotoxic when systemically administered. Despite classic pericarditis symptoms, the patient underwent an urgent coronary angiogram due to elevated cardiac troponin I level, EKG changes, and wall motion abnormalities on her echocardiogram. During her angiogram, it was found that she had multiple stenotic coronary artery lesions, with no acute total coronary occlusions, and percutaneous coronary intervention (PCI) was done with placement of a single drug-eluting stent for a 95% stenotic lesion in the left anterior descending artery. The patient was discharged after an uneventful hospitalization on dual antiplatelet therapy with aspirin and prasugrel, and colchicine for pericarditis. It is likely that the patient's presentation was the result of a perimyocardial inflammatory process secondary to intravesically administered mitomycin C, rather than an acute coronary syndrome. While the pathophysiological basis of cardiotoxicity of systemically administered mitomycin C is well documented, more studies are needed to determine whether intravesical MMC may cause cardiotoxicity.

## 1. Introduction

Acute pericarditis refers to inflammation of the pericardial sac—a tough double-layered fibroserous sac which covers the heart. Pericarditis has multiple causes, including infections, autoimmune diseases, metabolic disorders, neoplasms, trauma, radiation, and drugs [1–3], and its clinical manifestations typically include sharp and pleuritic chest pain that is improved by sitting up and leaning forward, a pericardial friction rub, EKG changes of new widespread ST elevations and PR depressions, and pericardial effusion [4]. Pericarditis is frequently self-limiting, and nonsteroidal anti-inflammatory agents remain the first-line treatment for uncomplicated cases [5]. We have here presented the case of a 64-year-old woman who developed acute pericarditis three days after intravesical instillation of mitomycin C chemotherapy after transurethral resection of bladder tumor (TURBT).

## 2. Case Presentation

A 64-year-old postmenopausal woman presented to the emergency department at 10 am in the morning with chest pain and shortness of breath of approximately 12 hours duration. Her medical history included right breast invasive ductal carcinoma with bilateral mastectomies in 2015, recently diagnosed bladder cancer with TURBT and intravesical mitomycin C instillation three days prior to current presentation, type 2 diabetes mellitus, hypertension, obstructive sleep apnea, chronic obstructive pulmonary disease, and prothrombin G20210A mutation. She had quit smoking 5 years ago, prior to that she had smoked 2 packs per day for 45 years. She was asymptomatic until about 10 pm the night before presentation, when she developed shortness of breath and sharp pleuritic chest pain while she was lying in bed. The patient reported that her chest pain was located in the substernal region, radiating to the back, aggravated by deep breaths and by lying down, and alleviated by sitting up and leaning forward. She said that she had taken some ibuprofen which had reduced the intensity of pain. She did endorse that she was also experiencing a less prominent pressure like sensation in addition to the sharp pleuritic type of pain. She denied fevers, chills, cough, diaphoresis, palpitations, dizziness, abdominal pain, heartburn, nausea or vomiting, flu-like symptoms, recent illnesses, or any history of chest pain in the past. Her family history was significant for her brother having an episode of myocardial infarction at age 55.

On detailed inquiry of the patient's history of bladder cancer, the patient reported that she was diagnosed in 2012 after undergoing cystoscopy for an episode of gross hematuria and was found to have a low-grade bladder cancer. She underwent transurethral bladder tumor resection and 1 cycle of intravesical BCG treatment in 2012 and was asymptomatic after that with regular follow-up with urology. On a surveillance cystoscopy performed by the patient's urologist in December 2016, a lesion was seen in the bladder, for which a biopsy was subsequently done which showed papillary hyperplasia and inflammation with no cancer. For this recurrence, the patient was planned for a cystoscopic transurethral bladder tumor resection and instillation of 40 mg of mitomycin C in the bladder, which she underwent three days before her current hospital presentation. The patient was intubated during this procedure which was uneventful, and was subsequently extubated without difficulty.

**Figure 1:**
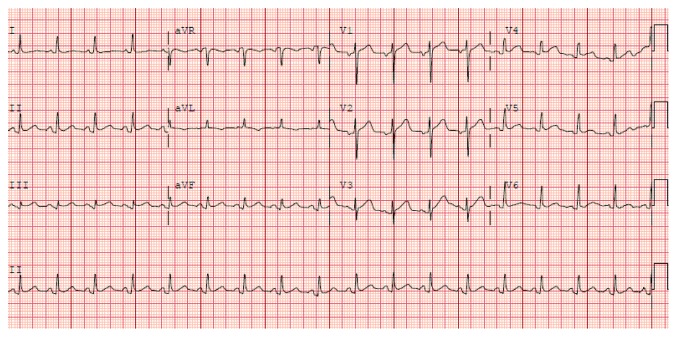
12-lead electrocardiogram obtained on arrival of the patient to the emergency department 3 days after undergoing TURBT with intravesical mitomycin C instillation showing diffuse concave ST elevations with PR depressions in all leads, except in aVR which showed ST depression and PR elevation.

The patient's oral temperature on presentation was 98.0 F, heart rate of 107/min, respiratory rate of 25/min, and blood pressure of 152/63 mmHg (right arm, supine position). On examination, the patient was in no acute distress and appeared comfortable, although was complaining of having mild chest pain, alleviated by leaning forward. Her laboratory data were as follows: cardiac troponins on the day of presentation, 0.57 ng/ml at 12:30 pm; D-dimer, 257 ng/ml; and white blood cell count, 12.2 K/ml with 67.3% neutrophils. 12-lead electrocardiogram (Figure 1) showed diffuse concave ST elevations with PR depressions in all leads, except in aVR which showed ST depression and PR elevation.

The patient was treated with aspirin 325 mg orally and nebulized albuterol. An echocardiogram was performed which showed normal left ventricular ejection fraction of 61–65%, mid and apical inferior wall motion abnormality, mild to moderate left ventricular diastolic dysfunction, and trivial pericardial effusion. Although the patient's clinical presentation and electrocardiogram findings were consistent with pericarditis, an urgent coronary angiogram was planned due to the possibility of the patient having an acute coronary syndrome, considering her continuing chest pain, elevated cardiac troponin level, echocardiogram findings of mid and apical inferior wall motion abnormality, and the presence of multiple risk factors of ischemic heart disease. The coronary angiogram, performed through right radial artery access, showed a normal left main stem artery, proximal left anterior descending luminal irregularities, a 95% stenotic focal lesion of the mid left anterior descending artery, 40% stenosis of distal left circumflex artery, 30% stenosis of the ostial right coronary artery, 40% stenosis of proximal right coronary artery, 50% stenosis of mid right coronary artery, and 99% stenosis of right posterior descending artery. Left ventricular diastolic pressure was recorded to be 24 mmHg. A single 2.5 × 20 mm synergy monorail drug-eluting stent was placed in the stenosed segment of the mid left anterior descending artery. The patient received 60 mg of prasugrel orally during the procedure, a bolus dose of intravenous tirofiban followed by a maintenance dose intravenous tirofiban drip, and was admitted to the telemetry floor. Dual antiplatelet therapy with aspirin 81 mg daily and prasugrel 10 mg daily was initiated, and the patient was also started on colchicine 0.6 mg every 12 hours for pericarditis. The remainder of the hospitalization was uneventful, and the patient was discharged on the third hospital day, with a plan for a staged percutaneous coronary intervention for the 99% stenotic lesion in the right posterior descending artery in a few weeks.

## 3. Discussion

The term “perimyocarditis” is used for cases of acute pericarditis that also demonstrate myocardial inflammation—it may be associated with increases in serum biomarkers of myocardial injury such as cardiac troponin I or T [6] and echocardiographic findings of diffuse/localized LV wall motion abnormalities [7, 8]. In clinical practice, it may be difficult to differentiate between perimyocarditis and ST elevation myocardial infarction due to EKG findings of ST elevation and cardiac biomarker elevation seen in both conditions [9, 10].

The patient's clinical presentation and EKG were strongly suggestive of pericarditis. Her chest pain was pleuritic and positional, with relief in a seated position and with ibuprofen—pointing more towards pericarditis than myocardial ischemia [1, 2]. The initial EKG obtained on presentation showed diffuse concave ST elevations with PR depressions in all leads, except in aVR which showed ST depression and PR elevation—a finding that is consistent with pericarditis [1, 2, 4]. Also, the distribution of the ST elevations on the EKG was more diffuse than what would be expected from her distal coronary lesions.

Due to her multiple risk factors—smoking, obesity, family history of early coronary artery disease, and a mildly elevated initial cardiac troponin I level of 0.57 ng/ml, an urgent echocardiogram was performed which showed wall motion abnormalities in the mid and apical inferior wall and trivial pericardial effusion. Coronary angiography performed subsequently showed a 95% focal stenotic lesion in the mid left anterior descending artery (LAD) and 99% lesion in the right posterior descending artery, with no acute total coronary occlusions. Since the patient had continued to complain of chest pain through her presentation—which may have been worsened due the tachycardia caused by pericardial pain combined with the stenotic lesion in the LAD resulting in a possible ischemic component to the pain (similar to an exercise stress test)—and considering the apical wall motion abnormality seen on the echocardiogram, a decision was made to proceed with percutaneous coronary intervention (PCI) with placement of a drug-eluting stent for the LAD stenosis. However, based on the pathophysiology of coronary artery disease leading to acute coronary syndromes, the underlying pathology in most cases of acute myocardial infarction is an acute intraluminal coronary thrombus formation within an epicardial coronary artery leading to total or near-total acute coronary occlusion, something which our patient did not have [11]. A repeat troponin I level which resulted after angiography was 0.54 ng/ml at 5:30 pm (a downtrend from the earlier level of 0.57 ng/ml at 12:30 pm). Given her 12 hours of continuous chest pain, this is a particularly low troponin I level if acute coronary syndrome was her primary issue [12].

**Figure 2:**
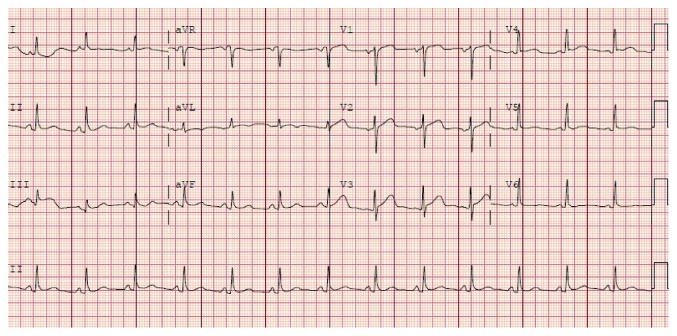
12-lead electrocardiogram obtained 15 hours after presentation showing persistence of the ST elevations with PR depressions in all leads, except in aVR which showed ST depression and PR elevation.

**Figure 3:**
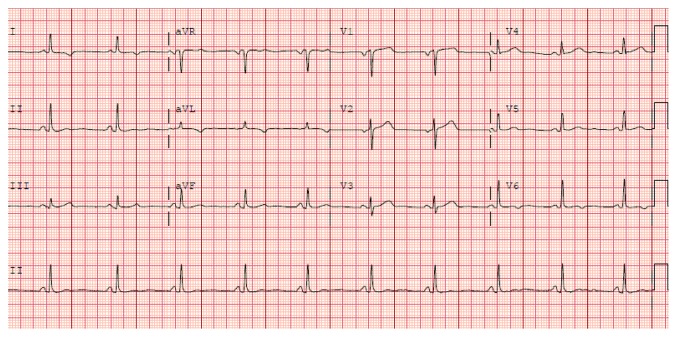
12-lead electrocardiogram obtained 3 weeks after hospital discharge showing return of PR and ST segments to baseline.

**Figure 4:**
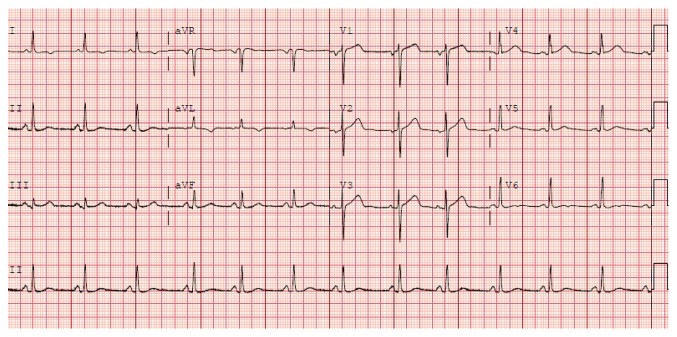
12-lead electrocardiogram obtained one week prior to the patient undergoing TURBT with intravesical mitomycin C instillation showing normal PR and ST segments in all leads.

An EKG obtained 15 hours after intervention (Figure 2) showed neither the resolution of the ST elevation nor the evolution of pathological Q waves, either of which should have occurred if ischemia was the reason for her EKG findings [13]. A follow-up EKG performed 3 weeks after hospital discharge (Figure 3) shows complete resolution of the EKG findings and was essentially unchanged when compared to her EKG performed 1 week prior to the TURBT (Figure 4). This is consistent with resolution of her pericarditis.

Mitomycin C is the most commonly used intravesical chemotherapeutic agent [14] and has an established role in intravesical administration following resection of low-risk non-muscle-invasive bladder cancer [15]. It exerts its biological effects by alkylation of cellular nucleophiles, including DNA, after undergoing enzymatic bioreduction into a bis-electrophilic intermediate [16]. A study was done to determine the systemic absorption of intravesical mitomycin C (MMC) in which fourteen patients with primary or recurrent non-muscle-invasive bladder cancer (NMIBC) underwent single early intravesical instillation of MMC (40 mg in 50 mL distilled water) immediately after TURBT. The results showed that maximal MMC plasma concentrations were reached 40 minutes after instillation with a highest plasma peak of 49.25 ng/ml. In the first samples, at 20 minutes after instillation, the plasma concentration of MMC was significantly correlated with the extent of TURBT (*P*=0.026) [17].

Side effects following intravesical administration of mitomycin C include myelosuppression [18], a self-limited chemical cystitis that rarely progresses to the shrunken contracted bladder [19], and a rash primarily involving the palms, soles, and genitalia, which is thought to be a manifestation of a hypersensitivity reaction [20]. While systemically administered mitomycin C has been known to be associated with dose-dependent cardiotoxicity, especially when combined with or given following doxorubicin [21], the association of cardiotoxicity with intravesically administered mitomycin C has not been studied. A study was done in 2013 which analyzed data retrospectively from 3 academic tertiary referral centers between 2000 and 2010, in which 47 patients (median age 70, range 32–85; 36 males and 11 females) who previously failed a median of 2 intravesical treatments, subsequently received 6 weekly treatments with sequential intravesical gemcitabine (1 g) and mitomycin C (40 mg) chemotherapy for non-muscle-invasive bladder cancer (NMIBC). Among the patients who developed complications as a result of the study, 1 patient was reported to have developed pericarditis shortly after the last treatment that was attributed to mitomycin C exposure [22].

## 4. Conclusion

The possibility of cardiotoxicity in the form of perimyocarditis occurring in association with intravesical mitomycin C instillation following TURBT has not been studied and with no documented case reports in existing literature and only one instance of pericarditis attributed to intravesical MMC instillation in a retrospective analysis [16]. While the pathophysiological basis of cardiotoxicity of systemically administered mitomycin C is well documented [14], more studies are needed to determine whether intravesical MMC may cause cardiotoxicity.
